# Neuroanatomical Reconstruction of the Canine Visual Pathway Using Diffusion Tensor Imaging

**DOI:** 10.3389/fnana.2020.00054

**Published:** 2020-08-18

**Authors:** Olivier Jacqmot, Bert Van Thielen, Alex Michotte, Johan de Mey, Steven Provyn, Jonathan Tresignie

**Affiliations:** ^1^Anatomical Research and Clinical Studies (ARCS), Vrije Universiteit Brussel, Brussels, Belgium; ^2^MOVE—HIM (Morpho Veterinary and Human Imaging) Brussels, Universitair Ziekenhuis Brussel (UZ Brussel), Brussels, Belgium; ^3^Department of Radiology, UZ Brussel, Brussels, Belgium; ^4^Odisee Brussel, Educational Department for Imaging Technologists, Brussels, Belgium; ^5^Anatomical Research, Training and Education (ARTE), Vrije Universiteit Brussel, Brussels, Belgium; ^6^Department of Neurology and Neuropathology, Neuroanatomy, UZ Brussel, Brussels, Belgium

**Keywords:** visual pathway, DTI, MRI, canine, stereotaxy

## Abstract

The first anatomical atlas of diffusion tensor imaging (DTI) of white matter pathways in the canine brain was published in 2013; however, the anatomical orientation of the entire visual pathway in the canine brain, from the retina to the cortex, has not yet been studied using DTI. In the present study, 3T DTI magnetic resonance (MR) images of three dogs euthanized for reasons other than neurological disorders were obtained. The process of obtaining combined fractional anisotropy and directional maps was initiated within 1 h of death. The heads were amputated immediately after MR imaging and stored in 10% formalin until dissection and histological sampling was performed. The trajectory of the visual pathway is dissimilar to the horizontal representation in other literature. To our knowledge, ours is the first study to visualize the entire canine visual pathway in its full antero-posterior extension. Fibers from the retina to the cortex passed through the optic nerve, optic chiasm, optic tracts, lateral geniculate nucleus, Meyer’s and Baum’s loops, and pretectal fibers. Their projections to the cortex were similar to those in the human visual pathway. The crossing of fibers at the optic chiasm occurred in 75% of fibers. In addition to advancing our knowledge in this field of study, these results could help plan neurosurgical and radiotherapeutic procedures to avoid unnecessary damage to the visual fiber system.

## Introduction

The human visual pathway consists of two neural tracts that traverse the brain in an axial plane. Optic nerve fibers connect the retina to the lateral geniculate nucleus (LGN) of the thalamus *via* the optic chiasm and optic tract, which is divided into the medial and lateral roots. The lateral root consists of neurons that synapse in the LGN and traverse through the retrolenticular portion of the posterior limb of the internal capsule to form the optic radiation in the visual cortex (Schuenke et al., 2010; Kamali et al., 2014).

The optic radiation fibers project to the ipsilateral visual cortex in the occipital lobe and are adjacent to the calcarine sulcus. Superior fibers follow a dorsomedial trajectory through the parietal lobe toward the occipital cortex, known as Baum’s loop, and inferior fibers follow an anteroinferior trajectory around the temporal horn of the lateral ventricle, turning medially and posteriorly to form Meyer’s loop. From there, they relay to the lingual gyrus of the calcarine sulcus of the occipital lobe (Kamali et al., 2014). A small number of optic nerve axons do not terminate on neurons in the LGN but continue along the medial root, forming the non-geniculate portion of the visual pathway. These axons pass through the superior colliculus and continue to the thalamus and brainstem, enabling the tracking of moving objects by unconscious eye and head movements. These axons may also terminate in the pretectal area where they are responsible for accommodation reflexes.

The exploration of white matter (WM) tracts in the brains of different specimens and species can explain behavioral and pathological characteristics (Jacqmot et al., 2017). Diffusion tensor imaging (DTI) provides a thorough anatomical overview of the white matter in terms of diffusion anisotropy and fiber orientation (Reinges et al., 2004; Yamada et al., 2008). The diffusion direction of fractional anisotropy can be traced across voxels to estimates fiber pathways (Basser et al., 2000). The acquisition time is a crucial element, as it determines the duration of the patient’s immobility during the sequence and consequently the quality of exploration (Kastler and Vetter, 2011). We have, therefore, elected to conduct postmortem animal experiments to improve image quality and avoid movement artifacts (e.g., breathing, vascular pulsations which can be the origin of ghost images) with an increased number of slices and total acquisition time of 5 h. However, when studying axonal architecture using DTI, it is very important to understand the limitations arising from the inhomogeneity of the water environment. First, the conventional DTI data acquisition and processing methods may not be able to properly handle a voxel containing more than one population of axonal tracts with different orientations. Second, DTI cannot provide information on cellular-level axonal connectivity. Multiple axons from individual cells may merge into or branch out from one voxel. Within a voxel, cellular-level axonal information of multiple compartments is averaged. The third important limitation is that afferent and efferent pathways of axonal tracts cannot be judged from the direction of water diffusion.

In human medicine, DTI is commonly used to study the anatomy of the normal brain and its maturation and aging (Dong et al., 2004). It can also be used as an aid for the diagnosis and prognosis of cerebral ischemia, multiple sclerosis, epilepsy, metabolic disorders, and brain tumors (Dong et al., 2004; Fernandez-Miranda et al., 2012). WM can be studied in detail using DTI. Furthermore, it shows a complete anatomical and statistical fiber atlas of WM (Wakana et al., 2004; Mori et al., 2008; Oishi et al., 2008), and can explain different neural regions, in combination with functional and anatomical MRI, functional connectivity (Hagmann et al., 2003), and histology (Bridge and Clare, 2006; Saleem and Logothetis, 2012; Mori et al., 2017; Castonguay et al., 2018). Using DTI, several studies have described the healthy human optic pathway (Staempfli et al., 2007; Hofer et al., 2010; Fernandez-Miranda et al., 2012; Zhang et al., 2012; Kamali et al., 2014; Rokem et al., 2017), Meyer’s loop asymmetries (Dreessen de Gervai et al., 2014), and healthy optic radiation (Sherbondy et al., 2008; Sun et al., 2013; Dayan et al., 2015). Some studies have examined WM/optic radiation integrity after glaucoma (Schoemann et al., 2014) and retinal injury (Ho et al., 2015), and observed optic radiation lesions (Klistorner et al., 2014).

Recently, researchers have adopted DTI in the study of WM tracts of the canine and feline brain (Jacqmot et al., 2013, 2017). In veterinary medicine, only anatomy books and histological examination of the optic pathway are available. Literature representation of the visual pathway in domesticated animals is largely inspired by that of human studies, where a horizontal (planiform) representation is described because the shape of the brain is gathered on itself. In most domesticated animal species, the cerebral hemispheres, cerebellum, and brain stem are arranged in a straight line. It is therefore useful to use DTI to have a 3D reconstruction of the complete visual pathway for veterinary use. Indeed in the future of veterinary medicine, the anatomical knowledge of the entire visual pathway could help plan neurosurgical and radiotherapeutic procedures to avoid unnecessary damage to the visual fiber system and could, therefore, enable the treatment of epilepsy and other brain surgeries or radiotherapies to the temporal lobes in the event of a tumor.

The present study aimed to provide a representation of the canine optic pathway anatomy using DTI and to confirm these findings *via* histological investigation.

## Materials and Methods

The methods used in the present study were following a previous study (Jacqmot et al., 2013). DTI images were obtained by echo-planar imaging (EPI) sequence and inherently involved various noises, such as eddy current distortion, head movement, and B0 inhomogeneity distortion. We, therefore, used a DTI preprocess script, which corrects and calculates the estimates of the diffusion tensor using the Functional Magnetic Resonance Imaging of the Brain Software Library (FSL) software. As this was not an experimental study, an ethics committee was not required to approve the study design.

### Animal Sampling

Three mesaticephalic dogs without neurological disorders [a male Poodle (age, 10 years; weight, 7.5 kg), a male Malinois shepherd (age, 4 years; weight, 30 kg), and a female Jack Russell terrier (age, 6 years; weight, 7 kg)] were euthanized for different reasons or known pathologies according to their owner’s will in a veterinary clinic. Immediately following euthanasia, animals were transported to the MRI unit of the University Hospital of the Vrije Universiteit Brussel. MRI scans were started within 1-h after postmortem, and it was concluded that there were no temporal differences between the samples at scanning onset. All the animals were placed in the dorsal decubitus position, orienting their heads in a ventrodorsal position.

### DTI MRI Acquisition and Directional Mapping

DTI MR images were obtained using a 3T Achieva (Philips Healthcare, Eindhoven, Netherlands) unit with a neurovascular 16-channel head coil and single-shot EPI sequence using the following parameters: repetition time (TR) = 7,855 ms; echo time (TE) = 55 ms; 2 excitations; field of view = 220 mm; slice thickness = 2.2 mm; number of stacks = 1; number of slices = 50; matrix = 112 × 109 zero-filled to 224 × 224; acquisition voxel size = 1.96 × 2.10 × 2.20 mm^3^; interpolated to 0.98 mm isotropic voxels; diffusion encoding in 46 directions, with *b* = 2,800 s/mm^2^. After this DTI MRI images were obtained, in addition to T2–weighted Turbo Echo Spin images (3000/80/32; TR/TE/excitations), 230 mm field of view, 1 mm sections, matrix 400 × 255 mm^2^ zero-filled to 512 × 512 matrices, acquisition voxel size 0.57 × 0.72 × 1 mm interpolated to 0.45 mm isotropic voxels. The total acquisition time was 5 h.

Although zero filling does not add any information to the input raw data, it can nevertheless improve the apparent spatial resolution of the image due to reduced partial volume artifacts. Zero filling acts as a method to interpolate the signals from neighboring voxels, giving the image a smoother and less “pixelated” appearance. The choice of 46 directions represented a somewhat arbitrary balance between accuracy in fitting the tensor model (increasing the number of encoding directions decreases the variance in the tensor model parameters) and the number of sections that could be acquired (our imaging system limits the number of images per series to 512).

We used T2-weighted images because it allows seeing water and fat while T1 only allows seeing fat. The use of freshly euthanized animals allowed us to use the T2 without using data correction for possible movement artifacts as is the case with anesthetized animals (Pieri et al., 2019).

### DTI Tractograms

**Figure 1:**
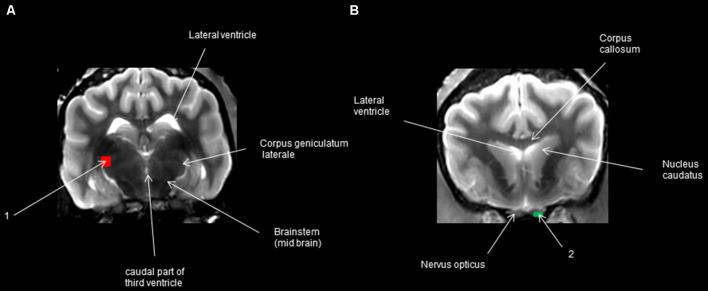
Regions of interest (ROIs) in transversal view. **(A)** The caudal transversal section at the 3rd ventricle behind the pituitary gland; **(B)** transversal section at the optic nerve in front of the optic chiasm.

The WM tracts were estimated using deterministic tractography (Jellison et al., 2004) using the MR Fiber Trak software (Philips Healthcare, Eindhoven, Netherlands) through the fiber assignment by continuous tracking algorithm (Mori and van Zijl, 2002; Jacqmot et al., 2013, 2017) with fractional anisotropy (FA) threshold of 0.15 and an angle threshold of 45°. Tracking was initiated from a start location or a seed point—in this case, a seed region of interest (ROI) in both forward and backward directions, defined by the major eigenvector at the seed point. Before using these points, we carefully examined the parameter settings to ensure that the tracked fibers were rational. Using the available tractography algorithm, we tested different fractional anisotropies, lengths, and angles, and the best results were obtained with the following parameters. The propagation was terminated when the tract trajectory reached a voxel with an FA less than 0.15 (the estimated eigenvector direction becomes less accurate as FA decreases and becomes very sensitive to image noise for FA less than 0.15), or when the tract was less than 10 mm, or when the angle between two consecutive steps was greater than 45°. A full set of fiber trajectories was obtained by placing seed points in all ROIs where an experienced veterinary neuroanatomist located the visual pathway (Figure 1), i.e., multiple ROIs were placed on the transversal plane. To start one multiple ROI was set on the midbrain rostral side at the lateral geniculate nucleus LGN (one on the left side and one on the right side) and two multiple ROIs were also placed on the optic nerve before the optic chiasm near the eyeballs (on the right side and the same on the left side), which enabled us to broaden the visualization of the optic tract. To reveal the bundles, the transversal plane was considered, and the stated ROIs were set. Smaller ROIs were used on enlarged images for better distinction and to avoid unwanted fibers. To help localize the positions for setting ROIs, T2-weighted high-resolution images were superimposed on the apparent diffusion coefficient map (ADC-map) to assist in the recognition of the connections between the different anatomical structures of the visual pathway. Fiber trajectories were displayed with color maps and were volume-rendered or overlaid onto the T2-weighted images. The visual pathways were recognized and correlated with histological descriptions, anatomical textbooks (Evans and Evans, 1993; Barone and Bortolami, 2004; Schuenke et al., 2010), and human DTI atlases and studies (Staempfli et al., 2007; Hofer et al., 2010; Fernandez-Miranda et al., 2012; Zhang et al., 2012; Kamali et al., 2014; Rokem et al., 2017).

It should be noted that we decided not to place any ROIs at the cortical center of vision (i.e., at the occipital pole) to avoid visualizing any fibers other than those of the visual pathway, such as the fronto-occipital and temporo-occipital fibers (Jacqmot et al., 2013).

### Gross Dissection and Histological Preparation

After performing the MRI-based examination of the brain, the dogs were decapitated, and the heads were immersed in a 10% formaldehyde solution to fix and harden the brain. After fixing for 2 months in the formalin solution, the skull was opened with a chisel, hammer, and gouge forceps, and the brains were sliced for histological study. Histology was used to confirm the presence of different fibers of the visual pathway at the level of the optic nerves, the optic tract, the LGN, the internal capsule, and the cortex and to make sure there were no signs of tissue deterioration during the 5 h of acquisition. Brains were cut into successive 5 mm thick slices in the transversal plane. Blocks of the frontal, temporal, parietal, and occipital cortices underlying the WM, hippocampus, and basal ganglia were embedded in paraffin for detailed histological analysis. Slices (6 μm thick) were stained with Masson’s trichrome, Klüver Barrera, and hematoxylin-eosin. The analysis by the Klüver Barrera method was carried out by OJ and AM (professor of neurology and anatomical pathology) and this type of staining allows the highlighting on sections of the presence of myelinated fibers, which allows us to confirm the presence of this type of fiber in the regions which have been revealed by the DTI technique. Representative photomicrographs were analyzed to illustrate the position and trajectory of the visual pathways.

## Results

**Figure 2:**
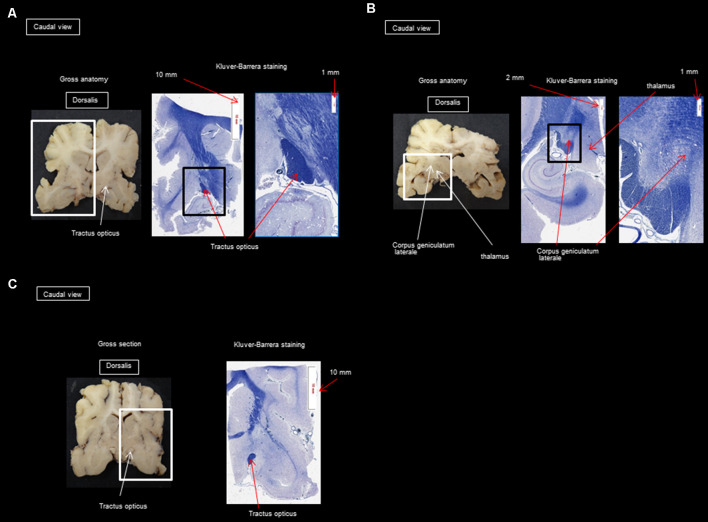
**(A)** Transversal section at the mammillary body; **(B)** transversal section at the thalamus and the lateral geniculate nucleus; **(C)** transversal section at the anterior commissure.

**Figure 3:**
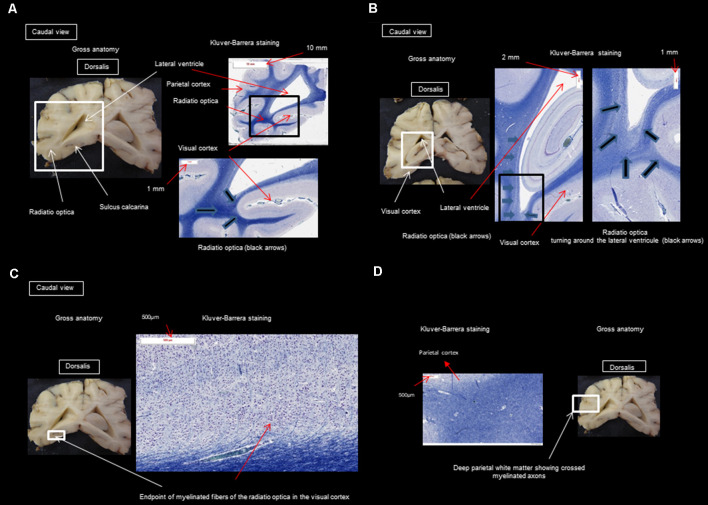
**(A)** Transversal section through the splenium of the corpus callosum and occipital visual cortex; **(B)** transversal section at the pulvinar nucleus and splenium of the corpus callosum; **(C)** transversal section through the splenium of the corpus callosum and occipital visual cortex; **(D)** transversal section through the splenium of the corpus callosum and parietal cortex.

Gross dissection was used to show where the histological sectioning was performed (Figures 2, 3). It should be noted that the results described below come from the three dogs mentioned in the section on “Materials and Methods” (“Animal Sampling” section).

**Figure 4:**
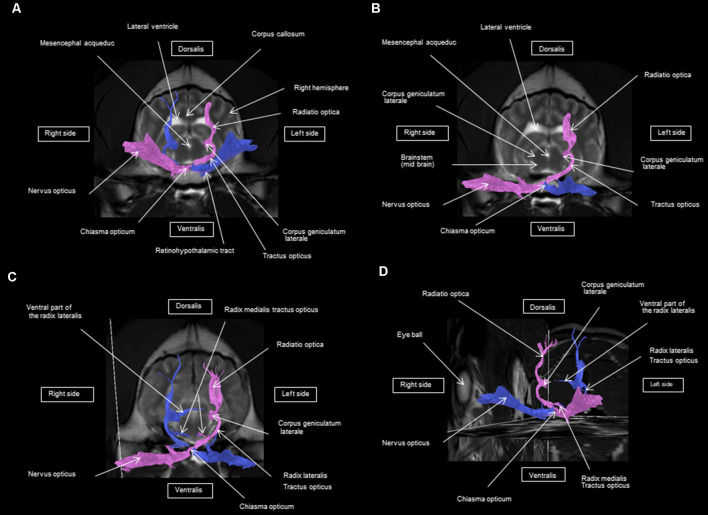
**(A–D)** Rostral-transversal view of the visual pathway.

**Figure 5:**
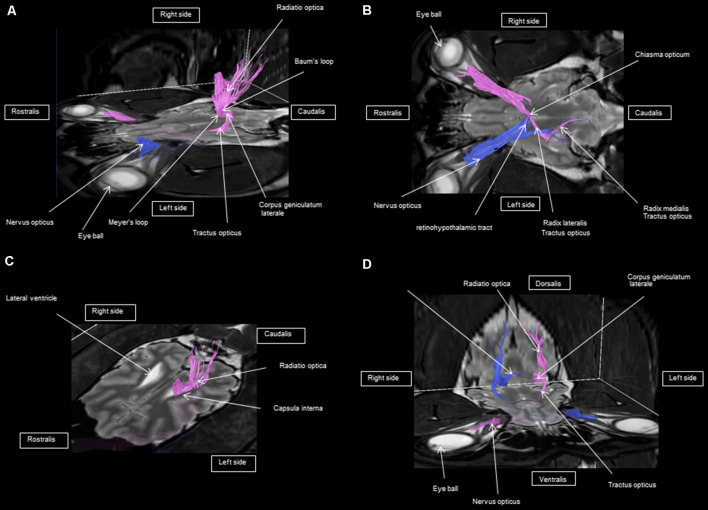
Frontal view of the visual pathway. **(A)** Laterodorsal view of the visual pathway. **(B)** Dorsal view of the optic tract, nerve, and chiasm. **(C)** Frontal view of optic radiation at the crus caudal internal capsule. **(D)** Craniofrontal view of the visual pathway.

**Figure 6:**
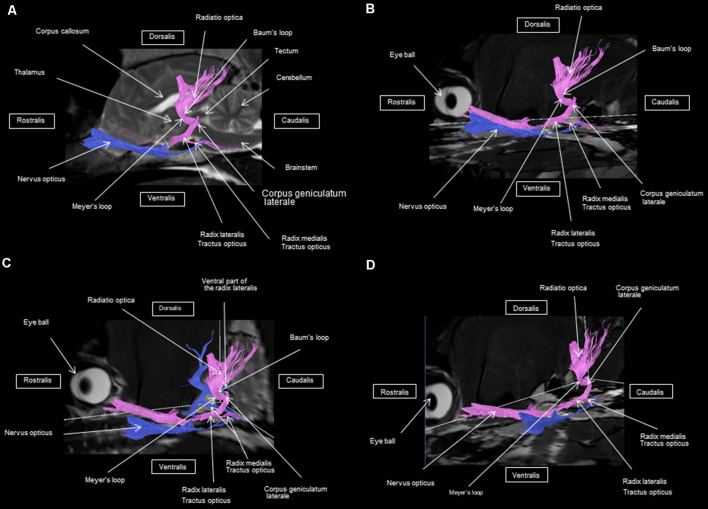
**(A–D)** Sagittal view of the visual pathway.

**Figure 7:**
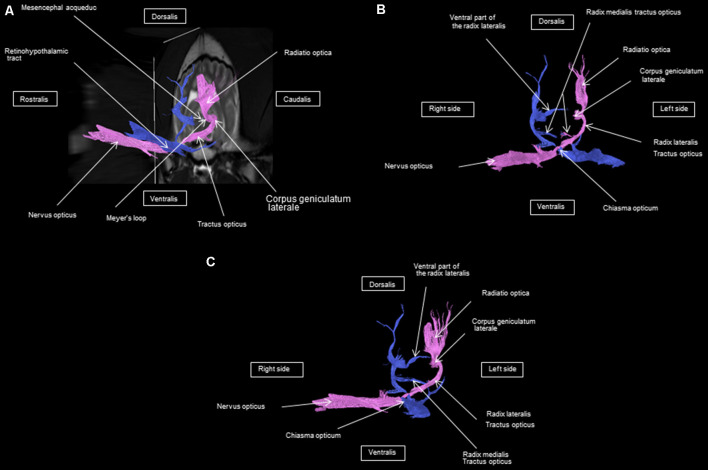
**(A)** Lateral view of the visual pathway. **(B)** Rostral view of the visual pathway. **(C)** Rostrolateral view of the visual pathway.

As shown in Figures 4–7, by setting one multiple ROI on the midbrain rostral side at the LGN and two multiple ROIs on the optic nerve before the optic chiasm, we obtained a bundle of fibers (pink) going from this nucleus to the optic nerve by crossing at the level of the chiasma (this enabled viewing of the entire visual pathway from the eyeballs to the cortex). Then we put another multiple ROI on the LGN on the other side on the same cross-section and we got a bundle of fibers (blue) going from the LGN to the optic nerve of the other eye, by crossing at the level of the chiasma.

The observed visual pathway begins at the retina, where fiber bundles forming the optic nerve are followed by the optic chiasm and tracts, which are divided into the medial and lateral roots (Figures 4–7). The medial root reaches the brainstem nuclei, particularly the eye motor muscle, head, and neck muscle nuclei (*via* tectal fibers), whereas the lateral root projects onto the LGN, forming a loop. The pretectal fibers are connected at the Edinger-Westphal nucleus of the pupil and enable miosis reflexes. Other fibers project through the internal capsule to form the optic radiation ending at the parietal and occipital cortices. Following the description of the different parts of the visual pathway (optic nerve, chiasma, optic tract, optical radiation, and projection at the cortical level), we note that the reconstruction in its entirety (from the retina to the cortex without interruption) reveals a helical structure.

Most of the fibers cross over at the optic chiasm; therefore, the left eye projects to the right hemisphere of the brain and right eye projects to the left. Two to three fibers join one eye to the other, continuing to form the retinohypothalamic tract (Figure 5B). It should be noted that the pink fiber bundle was first obtained by placing a multiple ROI on the LGN and the multiple ROI corresponding to the other side (blue) was then placed on the contralateral LGN. This is the reason why one has the impression that the fibers coming from the left retina seem to be broken compared to those of the right.

At the internal capsule, the rostrolateral fibers projecting onto the occipital cortex form Meyer’s loop, while the caudomedial fibers projecting onto the parietal cortex form the Baum’s loop; however, there are no fiber projections between these two cortices. Furthermore, Meyer’s and Baum’s loops are minimally delimited (Figures 5A, 6, 7).

We noted the presence of retinohypothalamic fibers in the optic nerve that terminates in the suprachiasmatic nucleus of the hypothalamus, the first relay to the epithalamus (Figure 5B).

## Discussion

MR imaging is a non-invasive technique used for exploring the brain. More specifically, DTI visualizes the different intracerebral connections based on the anisotropy of the water molecules in the myelin sheath and the cytoplasm of neurons (Basser et al., 2000; Bammer, 2003; Reinges et al., 2004; Yamada et al., 2008; Zhang et al., 2012). Neural tissue, especially white matter, is made up of densely aligned axons surrounded by oligodendrocytes (glial cells, producing myelin to “isolate” nerve cells and increase the rate of conduction of electrical depolarizations). The axons are frequently organized in more or less compact bundles. The movement of water molecules is significantly limited in the perpendicular direction and therefore flows more easily along the neural beams. Anisotropy can be influenced by variable factors such as the thickness, number, and density of axons, fiber consistency, and the thickness of myelin sheaths. Axonal transport, microtubules, and neurofilaments seem to play only a minor role in the anisotropy measured by MR imaging. The shape of the probability of displacement differs at each location of the brain according to local microarchitecture. It is therefore prudent to consider the diffusion MR imaging applied to brain tissue as an indirect measure of fiber direction and, to some extent, the integrity of myelin, remembering that myelin does not necessarily underlie the genesis of anisotropy, but potentiates it. Macroscopic anatomical gross dissection of the brain makes it difficult to find these intracerebral connections. Histological examination after staining allows the identification of neurons, but reconstruction using DTI provides a complementary 3D representation of the visual pathway and stereotaxic measurements. Contrary to what is illustrated in veterinary anatomy books, the visual pathway in dogs is not a horizontal structure, but rather a helical structure. From our observations, the path of the fibers from the retina to the LGN passes through the optic nerve, chiasm, and tracts, which is consistent with what is described in the literature in both animals and humans (Barone and Bortolami, 2004; Dyce et al., 2010; Schuenke et al., 2010).

It has been reported that the crossing of fibers at the chiasm level is at least 75% in dogs and that it can vary depending on the breed and individual (Brooks et al., 1999; Barone and Bortolami, 2004). In the current study, using the DTI method, we observed a crossing of more than 75%, which is similar to the 100% observed in Siamese cats and guinea pigs (Brooks et al., 1999; Barone and Bortolami, 2004).

In the optical tract, we observed a lateral root that projected to the dorsal and ventral components of the LGN and a medial root that projected to the rostral colliculus. These findings are consistent with what has been previously described (Barone and Bortolami, 2004). Also, the function of the different roots of the optical tract that has been described in earlier studies (Barone and Bortolami, 2004; Schuenke et al., 2010) agrees with our representation obtained by DTI. The dorsal portion of the lateral root of the optical tract is involved in visual perception. Its fibers project through the internal capsule and form the optical radiations, which are projected into the occipital and parietal cortices. In mammals, the ventral lateral root of the optical tract coordinates visual perception with the accommodation and movements of the eyes, which are controlled by the reflex pathways of the medial root of the optical tract. Medial root fibers follow the rostral colliculus, pretectal nucleus, and interstitial nucleus, defining the path of accommodation (Barone and Bortolami, 2004; Kamali et al., 2014). The medial root regulates the reflex pathway of somatic motor function. The projections of the colliculus rostral to the motor nuclei of the oculomotor and trochlear nerves are mainly homolateral and are heterolateral to the abducens nerve (Barone and Bortolami, 2004; Kamali et al., 2014).

In the present study, we also noted the presence of retinohypothalamic fibers in the optic nerve that terminates in the suprachiasmatic nucleus of the hypothalamus, the first relay to the epithalamus. The retinohypothalamic and retino-pretectal pathways are responsible for the analysis of the average luminance of the image for the registration of biological rhythms on the nycthemeral cycle and the photo motor reflex (Schuenke et al., 2010; Renard and Sellem, 2014).

In humans, at the LGN, a ribbon of fibers constitutes the optical radiation. They emerge from the LGN and pass through the retrolenticular part of the posterior limb of the inner capsule. The optical radiation fibers project into the ipsilateral visual cortex in the occipital lobe adjacent to the calcarine sulcus. These parallel superior fibers, Baum’s loop, extend dorsomedially through the parietal lobe to the occipital cortex. The lower fibers extend antero-inferiorly around the temporal horn of the lateral ventricle, turning inward and backward to form Meyer’s loop before relaying to the lingual gyrus of the calcarine sulcus of the occipital lobe (Hofer et al., 2010; Schuenke et al., 2010; Kamali et al., 2014). Our results show that in dogs, the fibers of Meyer’s loop pass near the temporal lobe to project themselves into the occipital cortex. The fibers of Baum’s loop make a caudomedial path to project at the level of the parietal cortex. The cortices were delineated in our study with the help of the literature (Barone and Bortolami, 2004; Dyce et al., 2010; Schuenke et al., 2010).

Most existing methods to diagnose vision pathologies focus mainly on the retina, whereas an important part of the visual system is represented by the visual pathways in the brain. Advances in neuroimaging, in particular DTI, allow the identification and characterization of WM fibers. This is particularly highlighted in studies on fiber degeneration caused by open-angle glaucoma (El-Rafei et al., 2013; Schoemann et al., 2014), excitotoxic retinal injury (Ho et al., 2015), multiple sclerosis associated with optic radiation lesions, or inflammatory damage of the posterior visual pathway (Gabelic et al., 2013; Klistorner et al., 2014).

To our knowledge, there are no similar studies that have been conducted in veterinary medicine. Our present study has allowed a complete 3D reconstruction of the visual pathway in animals, specifically dogs. For the first time, the helical anatomy of the visual pathway has been revealed, as well as the identification of Meyer’s and Baum’s loops, which have been identified in humans, but not in animals. The identification of Baum’s and Meyer’s loops is important because, in human medicine, clinical observations indicate that Meyer’s loop contains fibers representing both ipsilateral and contralateral retinal halves. Injury to this fiber bundle inevitably leads to homonymous hemianopia of the upper quadrant, a particular type of partial blindness (Nilsson et al., 2007; Hofer et al., 2010). The ability to identify and delimit Meyer’s loop is important to administer neurosurgical interventions of the temporal lobe, which are currently used for the treatment of drug-resistant epilepsy of the temporal lobe (Yamamoto et al., 2007; Dreessen de Gervai et al., 2014). Since normal variations in the anatomy of Meyer’s loop have been reported in previous studies, knowledge of the exact extension of Meyer’s loop is essential to avoid postoperative visual impairments after temporal lobectomy. It is therefore important to visualize and identify all parts of optical radiation in each patient to preserve optical radiation during brain surgery (Hofer et al., 2010).

Histology was used only to confirm the presence of different fibers of the visual pathway in the optic nerves, optic tract, LGN, internal capsule, and cortex. The staining by Klüver Barrera method was used to highlight myelin (commonly used stain to observe myelin under light microscopy) and this information was useful to us to confirm the presence of myelinated axons in the same place as those observed during reconstruction by the DTI technique. The DTI technique allows knowing what type of fibers we are dealing with by the use and precise placement of ROIs. Another technique is the Klingler method [a research method which allows understanding the imagery given by the tracking of the white fibers of the brain (N’Dri Oka et al., 2007)], but its major drawback is the long preparation time and arduous dissection. Also, the retraction of the brain only allows approximate measurements (it is not known precisely whether they are fibers of the same bundle).

In addition to using histology to confirm that there are limited tissue deterioration and chemical reaction, it has also been reported that diffusion tensor imaging of live and fixed brains provides similar results (Sun et al., 2005). This reveals three important facts: (1) water molecules move, even in postmortem brains, unless the sample is frozen; (2) DTI uses this water motion as a probe to infer the neuroanatomy; and (3) the information DTI carries is dominated by static anatomy and is less influenced by physiology.

The strength of our tracing lies in the fact that by placing one multiple ROI on the LGN, the reconstruction was done from the LGN to the retina and the cortex at the same time. This greatly limits the appearance of foreign fibers on this route.

A limitation of our present study is that these results can be improved because the technique used has its limits (some fibers might be missed due to the limited technology). The diffusion tensor model works well in regions where there is only one population of fibers. On the other hand, its performance decreases when the region contains several populations of fibers such as the semi-oval centrum where the cortico-spinal tract, the corpus callosum, and the arched bundle intersect. Indeed, DTI does not solve the “fiber crossover problem” (PCF; Hagmann et al., 2006). If two nerve fibers cross in a given voxel, the resolution of this conflict depends greatly on the type of data processing and the algorithm applied for tractography. The PCF can artificially modify the anisotropy. Methods with higher angular resolutions (HARDI) however make it possible to overcome this problem (Hagmann et al., 2006, 2010). This problem can also be resolved by working with more complex modeling of the fibers within the voxels [Neurite Orientation Dispersion and Density Imaging (NODDI); Zhang et al., 2012]. The displacement in the brain of water molecules is largely dependent on the cellular organization of the studied tissue and the use of a mathematical model called NODDI makes it possible to analyze the diffusion MR imaging data to determine the microstructure of the studied tissue (Sarrazin et al., 2019). For diffusion spectrum imaging, which is an improvement of HARDI, the acquisition time is extended, because a greater number of diffusion gradients are necessary (>120, ideally 515 or more) to obtain this high resolution. This method allows us to overcome a variety of difficulties encountered with DTI and makes it possible to generate connectivity maps closer to reality.

Another limitation is that the 3D reconstruction was performed on freshly euthanized animals with an acquisition time of 5 h. The use of freshly euthanized subjects allowed us to avoid artifacts related in particular to respiratory movements. Also, the use of one multiple ROI on the LGN enabled the observation of the complete visual pathway and two other multiple ROIS were put on the optic nerve to increase the number of fibers because if we had put multiple ROIs at the level cortex, for example, we would have generated fibers which would not constitute the visual pathway like the occipitofrontal, parieto temporal fibers. It would be interesting to repeat our experiment using live animals and in a clinical routine, similar to Anaya García et al. (2015), for our previous study of the canine corticospinal tract (Jacqmot et al., 2013), and WM in the sheep brain (Pieri et al., 2019). Further, there are three types of skulls, mesaticephalic, brachycephalic, and dolichocephalic. We carried out our experiments on mesaticephalics of different age, sex, and breed. Therefore, we observed that the complete visual pathway was similar in the three individuals used in our study. It would, however, be interesting to carry out this study in the other two types of skulls to see if the visual pathway has the same spatial arrangement. This study was, therefore, a first visualization of the complete visual pathway using the DTI technique in dogs and provided valuable information for didactic, clinical, and research purposes.

## Conclusion

To our knowledge, this is the first study to visualize the entire canine visual pathway in its full antero-posterior extension. It is necessary to repeat our experiment on live animals and in clinical routine to investigate the integrity of the visual pathway in pathologies such as glaucoma and multiple sclerosis. DTI ensures the reconstruction of anatomically correct fiber bundles of optic nerves, optic chiasm, Meyer’s and Baum’s loops, pretectal fibers, and their projections at the cortex. In addition to advancing our knowledge in this field of study, these results could help plan neurosurgical and radiotherapeutic procedures to prevent unnecessary damage to the visual fiber system.

## Data Availability Statement

All datasets presented in this study are included in the article.

## Ethics Statement

Ethical review and approval was not required for the animal study because as this was not an experimental study, an ethics committee was not required to approve the study design. Written informed consent was obtained from the owners for the participation of their animals in this study.

## Author Contributions

OJ, BV, SP, and JT contributed to the conception and design of the study. OJ, BV, and JM acquired the data. OJ wrote the first draft of the manuscript. OJ and AM wrote sections of the manuscript. All authors contributed to the article and approved the submitted version.

## Conflict of Interest

The authors declare that the research was conducted in the absence of any commercial or financial relationships that could be construed as a potential conflict of interest.

## Abbreviations

DTI: diffusion tensor imaging
EPI: echo planar imaging
FA: fractional anisotropy
LGN: lateral geniculate nucleus
NODDI: Neurite Orientation Dispersion and Density Imaging
ROI: region of interest
WM: white matter.
